# *In situ* addition of graphitic carbon into a NiCo_2_O_4_/CoO composite: enhanced catalysis toward the oxygen evolution reaction†

**DOI:** 10.1039/c9ra05195c

**Published:** 2019-08-12

**Authors:** Srinivasa N., Shreenivasa L., Prashanth S. Adarakatti, Jack P. Hughes, Samuel J. Rowley-Neale, Craig E. Banks, Ashoka S.

**Affiliations:** Department of Chemistry, School of Engineering, Dayananda Sagar University Bangalore India ashok022@gmail.com +91 8049092924; PG Department of Chemistry, KLE's PC Jabin Science College Hubballi India; Faculty of Science and Engineering, Manchester Metropolitan University Chester Street Manchester M1 5GD UK c.banks@mmu.ac.uk S.Rowley-Neale@mmu.ac.uk http://craigbanksresearch.com +44 (0)1612476831 +44 (0)1612471196; Manchester Fuel Cell Innovation Centre, Manchester Metropolitan University Chester Street Manchester M1 5GD UK

## Abstract

We present a rapid, environmentally benign one-pot synthesis technique for the production of a NiCo_2_O_4_/CoO and graphite composite that demonstrates efficient electrocatalysis towards the Oxygen Evolution Reaction (OER), in 1.0 M KOH. The NiCo_2_O_4_/CoO/graphitic carbon composite that displayed optimal OER catalysis was synthesized by nitrate decomposition in the presence of citric acid (synthesized glycine and sucrose variants displayed inferior electro kinetics towards the OER). Screen-printed electrodes modified with *ca*. 530 μg cm^−2^ of the citric acid NiCo_2_O_4_/CoO/graphite variant displayed remarkable OER catalysis with an overpotential (*η*) of +323 mV (*vs.* RHE) (recorded at 10 mA cm^−2^), which is superior to that of IrO_2_ (340 mV) and RuO_2_ (350 mV). The composite also exhibited a large achievable current density of 77 mA cm^−2^ (at +1.5 V (*vs.* RHE)), a high O_2_ turnover frequency of 1.53 × 10^−2^ s^−1^ and good stability over the course of 500 repeat cycles. Clearly, the NiCo_2_O_4_/CoO composite has the potential to replace precious metal based catalysts as the anodic material within electrolysers, thereby providing a reduction in the associated costs of hydrogen production *via* water splitting.

## Abstract 2

A facile synthesis technique for the production of NiCo_2_O_4_/CoO and graphite composites that demonstrate efficient electrocatalysis towards the oxygen evolution reaction.

## Graphical abstract

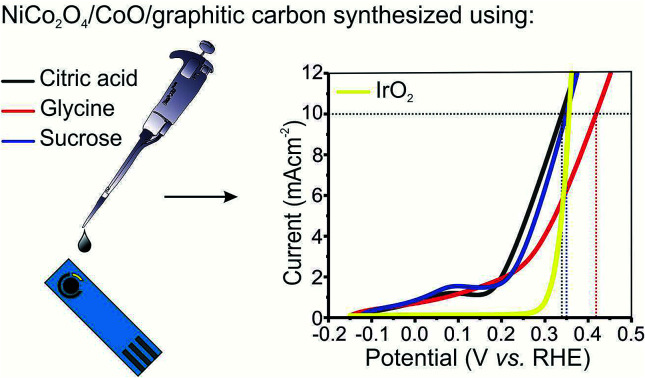

## 1 Introduction

The implementation of hydrogen as a clean fuel source has prompted the research and development of novel materials explored towards electrolytic water splitting, where proficient electrocatalysts will increase efficiency and economic viability of hydrogen production. The efficiency of overall water splitting is limited by the sluggish kinetics of the oxygen evolution reaction (OER), which is the anodic reaction within an electrolyser. The OER is a four step electron transfer (4OH^−^ → O_2_ + 2H_2_O + 4e^−^).^1,2^ Iridium and ruthenium oxide based catalysts^2–4^ are widely considered the optimal performing electrocatalysts towards the OER. However, the practical applications of these oxide catalysts are limited owing to their scarcity, high cost and catalytic instability. This has created an impetus to develop efficient, low cost and abundant catalysts for the OER.^5^

Table 1 summarizes studies that have explored cobalt and nickel based spinel oxides towards the OER.^6^ Good long-term stability in an alkaline medium and proficient electrochemical performance towards the OER make cobalt and nickel based spinel oxides interesting alternatives to precious metal based materials. However, their poor electrical conductivity hinders their large-scale application within industry. In order to mitigate poor electrical conductivity the NiCo_2_O_4_ is typically gelled with metals,^7–9^ and carbon based materials, such as carbon nanotubes,^10,11^ carbon fibers, carbon nanowires,^12–14^ graphene,^15^ and graphene aerogels.^16–19^ The gelling of graphene with NiCo_2_O_4_ uses harsh chemicals and usually takes prolonged durations of time, potentially several hours or days.^20^ Despite progress in the development of NiCo_2_O_4_ based catalysts, the preparation of cheap and environmentally benign materials with good electronic transport properties, high mechanical stability and good electro-kinetic ability towards the OER remains a challenge.

**Table 1:** Comparison of overpotentials and Tafel analysis, of the present study, at room temperature for reported NiCo_2_O_4_ based electrocatalysts^a^

Catalyst	Method of preparation and conditions used	Overpotential (mV)	Tafel slope (mV dec^−1^)	Ref.
NiCo_2_O_4_/CNT	Hydrothermal synthesis at 100 °C10 h followed by calcination 400 °C/2 h	340	133	22
NiCo_2_O_4_-NN	Hydrothermal at 90 °C/10 h and annealed in air at 320 °C/2 h	365	292	28
NiCo_2_O_4_-NS	Hydrothermal at 90 °C/10 h and annealed in air at 320 °C/2 h	415	393	28
NiCo_2_O_4_/VN800 Vulcan XC-72	Precipitation annealed 200 °C/3 h	385	75.7	30
NiCo_2_S_4_ NS/CC	Hydrothermal at 120 °C/8 h followed by annealed at 450 °C/2 h	260	123	31
Ni_0.33_Co_0.67_MoS_4_/CFC	Hydrothermal at 110 °C/6 h	283	68.8	32
NiCo_2_O_4_/CoO/graphitic carbon composite	Nitrate decomposition method at 500 °C/3 minutes	323	118	This work

a CNT – carbon nanotube; CFP – carbon fiber paper; NS – nano sheets; NW – nanowire; NN – nano needles; CC – carbon cloth; CFC – carbon fiber cloth.

To address these limitations when using NiCo_2_O_4_ based catalysts we describe a short (3 minute) one-pot wet chemical synthesis technique for a NiCo_2_O_4_/CoO composite that has varied amounts of incorporated graphitic carbon. The quantity of the graphitic carbon present in the NiCo_2_O_4_/CoO has been varied *in situ* using citric acid, sucrose and glycine precursors during the course of the reaction. The graphitic carbon in the proposed composite acts like 2D graphene in terms of providing an anchoring framework and enhancing the conductivity. The prepared NiCo_2_O_4_/CoO/graphitic carbon composite is thoroughly characterized by powder XRD, FTIR and SEM. The graphitic carbon rich NiCo_2_O_4_/CoO composite is then fabricated upon the surface of screen-printed electrodes (SPE) which are explored towards the OER in 1 M KOH. The NiCo_2_O_4_/CoO SPEs deliver an impressively low over potential of 323 mV at a current density of 10 mA cm^−2^, which is superior to the overpotentials reported in previous studies using traditional catalysts; IrO_2_ (340 mV) and RuO_2_ (350 mV)^21^ and Au/NiCo_2_O_4_ nano rod array (360 mV).^22^

## 2. Experimental section

### 2.1. Chemicals

Analytical reagent grade nickel nitrate hexahydrate (Ni(NO_3_)_2_·6H_2_O), cobalt nitrate hexahydrate (Co(NO_3_)_2_·6H_2_O), citric acid (C_6_H_8_O_7_), sucrose (C_12_H_22_O_11_), glycine (C_2_H_5_NO_2_), and potassium hydroxide (KOH) were purchased from SD Fine Chemicals Ltd. India and used as received without any further purification.

### 2.2. NiCo_2_O_4_/CoO/graphitic compound synthesis

**Fig. 1:**
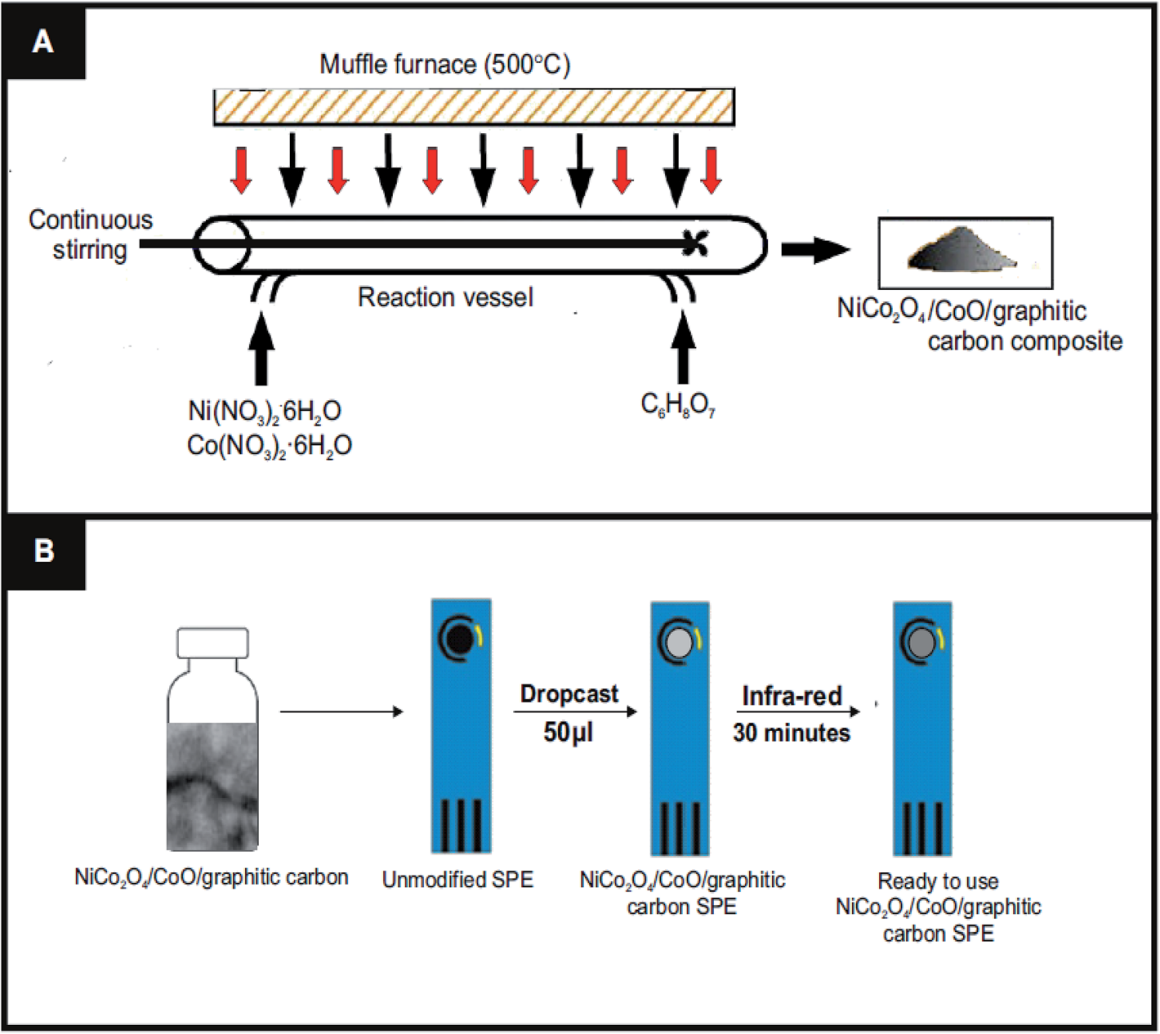
(A) The synthesis of the NiCo_2_O_4_/CoO/graphitic carbon composite; including reagents and conditions and (B) the steps required in order to fabricate SPE's with the NiCo_2_O_4_/CoO/graphitic carbon composite, post sonication.

The NiCo_2_O_4_/CoO/graphitic carbon composites were prepared using facile one-pot nitrate decomposition in the presence of citric acid, sucrose and glycine, Fig. 1(A) depicts the one-pot synthesis. In a typical synthesis, 0.859 mM of nickel nitrate hexahydrate (0.25 g) and 1.71 mmol of cobalt nitrate hexahydrate (0.50 g) were dissolved in 7 ml of deionized water and stirred until the metal nitrates dissolved completely. To the resulting solution, 1.432 mmol of citric acid (0.300 g) was added and continually stirred to get a uniform solution. Then, the precursor solution was kept in a preheated muffle furnace maintained at 500 °C. The reaction was rested to retain graphitic carbon in the prepared NiCo_2_O_4_/CoO composite by taking out the reaction vessel after 3 minutes from the preheated muffle furnace. Finally, the obtained powder NiCo_2_O_4_/CoO/graphitic carbon composite was crushed in a mortar and pestle and used for further study. Similarly, sucrose and glycine have been used to retain different amounts of graphitic carbon in the NiCo_2_O_4_/CoO.

### 2.3 Characterization equipment

The powder X-ray diffraction (XRD) pattern of the synthesized NiCo_2_O_4_/CoO/graphitic carbon composite was recorded using PANalytical X'pert PRO X-ray diffractometer with a graphite monochromatized CuKα radiation source. The Fourier-transform infrared (FTIR) spectrum of the NiCo_2_O_4_/CoO/graphitic carbon composite was recorded using IS5-800 Fourier transform infrared Nicolet spectrometer in the range of 400–4000 cm^−1^ in the transmittance mode. The morphology of the NiCo_2_O_4_/CoO/graphitic carbon composite was studied using a JEOL-5600LV scanning electron microscope (SEM). Transmission electron microscopy (TEM) images were obtained using a 200 kV primary beam under conventional bright-field conditions. The sample was dispersed onto a holey-carbon film supported on a 300 mesh Cu TEM grid. The electrochemical studies were carried out using a SP150 Biologic potentiostat.

### 2.4. Electrode preparation

The fabrication of the screen-printed electrodes (SPE's) utilized within this study is illustrated in Fig. 1(B), where 3 mg of the prepared NiCo_2_O_4_/CoO/ graphitic carbon composite was dispersed in 4 ml of deionized water under sonication for 30 minutes to get a homogenous dispersion. 50 μL of the resultant homogenous dispersion was drop casted onto an SPE and subjected to drying under infra-red light for 30 minutes. Thus, the electrocatalytic performance towards the OER of each modified SPE was ready to be assessed. Note the surface coverage of catalyst upon the SPE was *ca*. 530 μg cm^−2^.

### 2.5. Electrochemical measurements

Cyclic voltammetric (CV) and linear sweep voltammetric (LSV) measurements were carried out using a three-electrode system where the NiCo_2_O_4_/CoO/graphitic carbon composite SPEs, nickel and Ag/AgCl electrodes were used as working, counter and reference electrodes, respectively. The catalytic activity of the NiCo_2_O_4_/CoO/graphitic carbon composite towards the oxygen evolution reaction (OER) was studied in 1 M KOH, of pH = 14, in the potential range 0–1.5 V at a scan rate of 20 mV s^−1^.

## 3. Result and discussion

### 3.1. Physicochemical characterization of the NiCo_2_O_4_/CoO composites

**Fig. 2:**
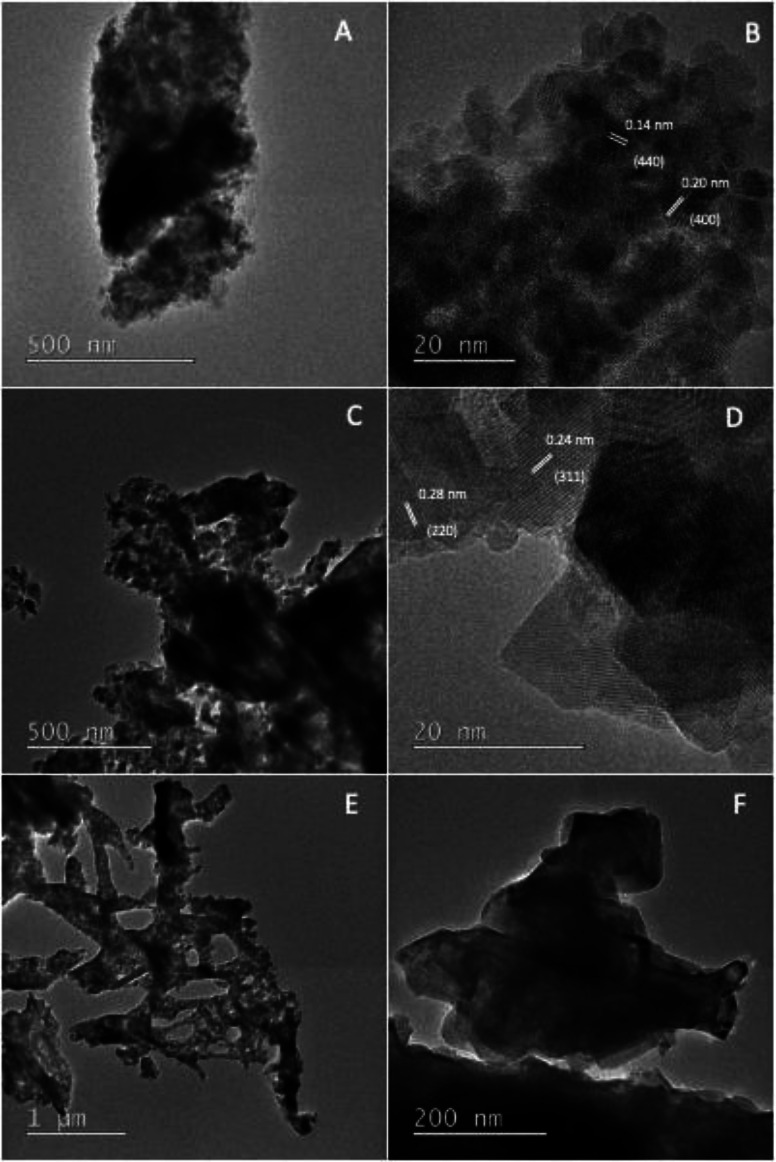
The TEM images of the NiCo_2_O_4_/CoO composite prepared at 500 °C/3 minutes by using (A and B) citric acid, (C and D) sucrose and (E and F) glycine.

The TEM images of the NiCo_2_O_4_/CoO nano-composite prepared by using citric acid are shown in Fig. 2(A and B), wherein the NiCo_2_O_4_/CoO nanoparticles (crystalline particles) are uniformly distributed and covered by a graphitic carbon network (amorphous region). The TEM images of the NiCo_2_O_4_/CoO nano-composite prepared by using sucrose (Fig. 2(C and D)) reveal the presence of randomly distributed agglomerated particles. The TEM images of the NiCo_2_O_4_/CoO nano-composite prepared by using glycine exhibit highly agglomerated particles with irregular morphology (Fig. 2(E and F)). The interplanar spacing's are highlighted in Fig. 2(B and D) where the spacing's of 0.14, 0.20, 0.24 and 0.28 nm are indexed to the (440), (400), (220) and (311) crystal planes of NiCo_2_O_4_, respectively.^23^

**Fig. 3:**
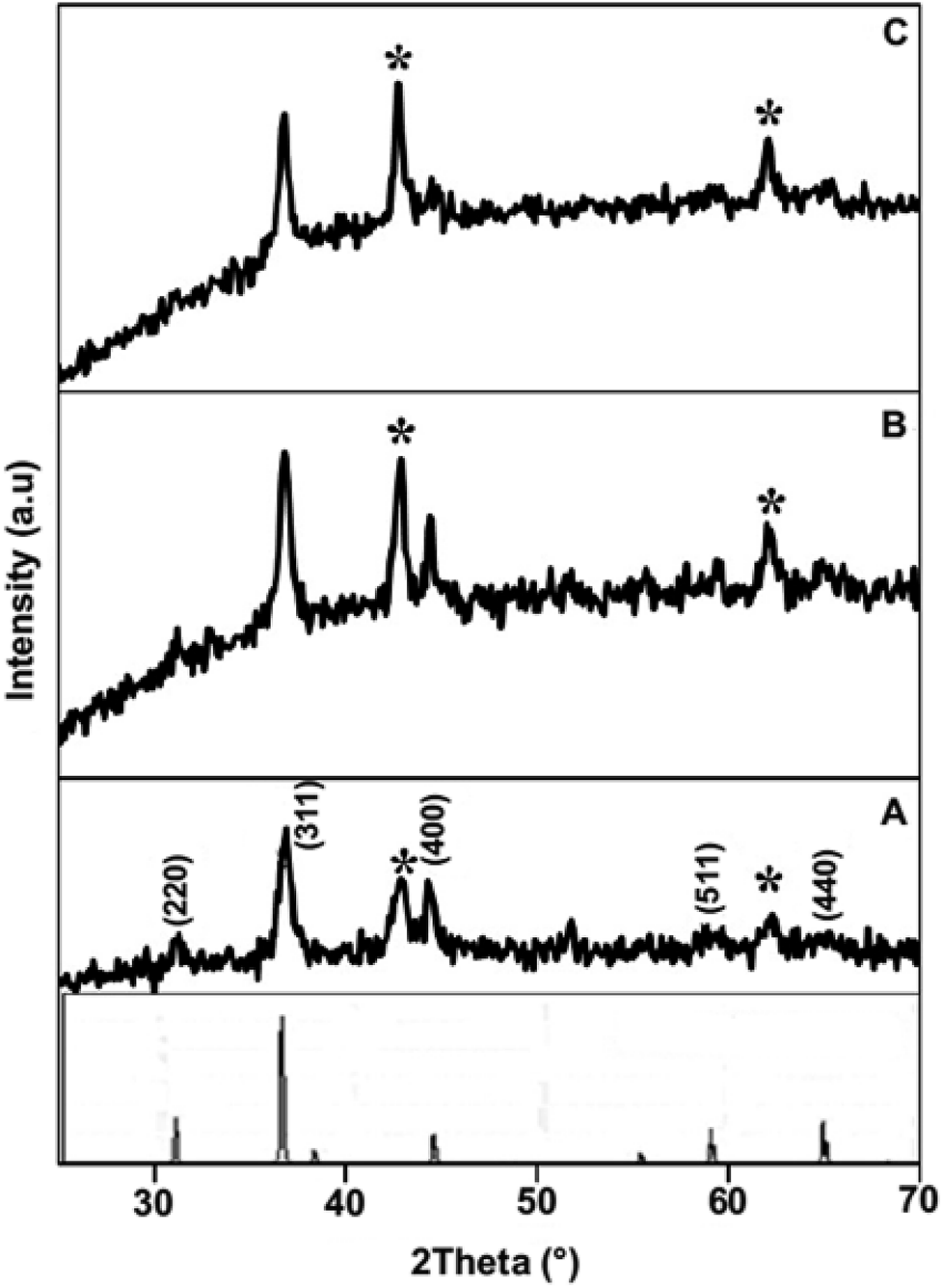
Powder XRD patterns of the NiCo_2_O_4_/CoO composite prepared at 500 °C/3 minutes by using (A) citric acid, (B) sucrose and (C) glycine.

**Fig. 4:**
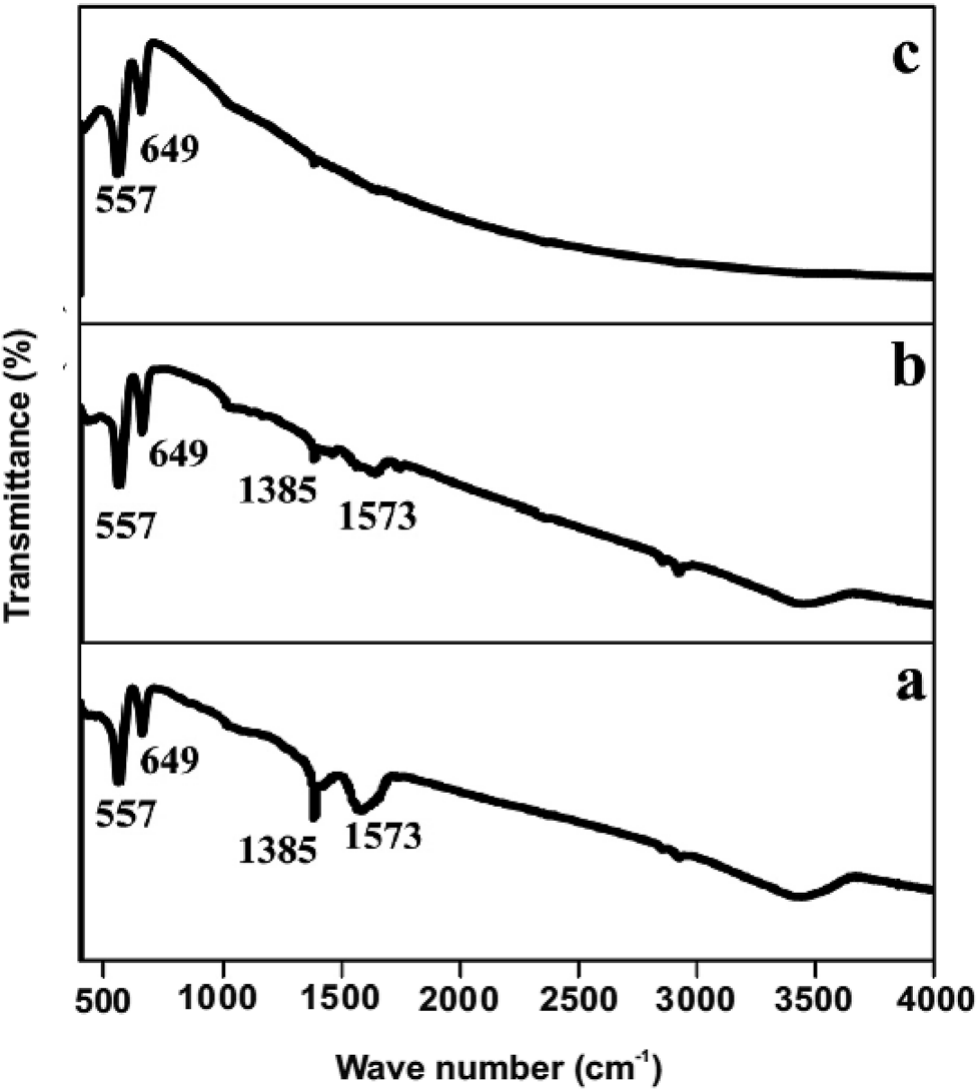
FTIR spectrum of the NiCo_2_O_4_/CoO composite prepared at 500 °C/3 minutes by using (a) citric acid, (b) sucrose and (c) glycine.

The crystallographic information of the synthesized product was analyzed using powder XRD measurements. The NiCo_2_O_4_/CoO composite with varied amounts of graphitic carbon is prepared using citric acid, sucrose and glycine where XRD patterns of each are presented in Fig. 3. The composites prepared using citric acid, sucrose and glycine exhibit peaks at 31.1°, 36.59°, 44.62°, 59.14° and 65.15° corresponding to the (220), (311), (400), (511), and (440) crystalline planes of the spinel cubic structure of NiCo_2_O_4_ with *Fd*3̄*m* space group (JCDS 73-1702). Additionally, the reflection peaks at 42.85° and 62.2°, indicated by *, show the presence of CoO. Fig. 4 indicates that the prepared product is composed of the NiCo_2_O_4_/CoO composite. FTIR analysis of the NiCo_2_O_4_/CoO composite can be seen in Fig. 4, it reveals the presence of graphitic carbon in the prepared NiCo_2_O_4_/CoO composite. The NiCo_2_O_4_/CoO composite prepared by using citric acid, sucrose and glycine, exhibit characteristic peaks at 557 cm^−1^ and 649 cm^−1^ assigned to the intrinsic metal–oxygen stretching vibrations in NiCo_2_O_4_/CoO composites.^24^ The peaks at 557 cm^−1^ and 649 cm^−1^ are assigned to the Co–O vibrational mode at the octahedral site and the Ni–O vibration mode at the tetrahedral site, respectively.^25^ Additionally, the NiCo_2_O_4_/CoO composite prepared using citric acid and sucrose exhibits a well-defined D band at 1385 cm^−1^, and a G band at 1573 cm^−1^ corresponding to the disordered carbon and ordered graphitic carbon, respectively.^26^ Furthermore, the composite prepared using citric acid possesses a higher quantity of graphitic carbon, confirmed with the intensity of the peak at 1385 cm^−1^, when compared to the composite prepared using sucrose. The NiCo_2_O_4_/CoO composite prepared using glycine does not exhibit peaks at 1385 cm^−1^ and 1573 cm^−1^ indicating the absence of carbon. Thus, the amount of the graphitic carbon in the prepared NiCo_2_O_4_/CoO composites varied in the order of citric acid > sucrose > glycine.

The catalytic activity of the synthesized NiCo_2_O_4_/CoO/graphitic carbon composite material could be largely dependent on the morphology. Hence, ESI Fig. S1† displays the SEM images of the prepared composite revealing that the particles are aggregated and thus appear like ledges with randomly distributed grains, large lateral dimensions and thin flat surfaces, where, a higher number of edge and active sites will result in greater OER performance. Careful observation reveals that the NiCo_2_O_4_/CoO/graphitic carbon composite prepared from glycine exhibits larger sized ledges compared to the composite prepared from citric acid and sucrose. Thus, it is expected that the composite prepared using citric acid and sucrose will exhibit better OER performance owing to the presence of graphitic carbon, increasing the number of electrical pathways, and high lateral size with thin and flat surfaces.

### 3.2. Application of the NiCo_2_O_4_/CoO composite SPE's towards the OER

**Fig. 5:**
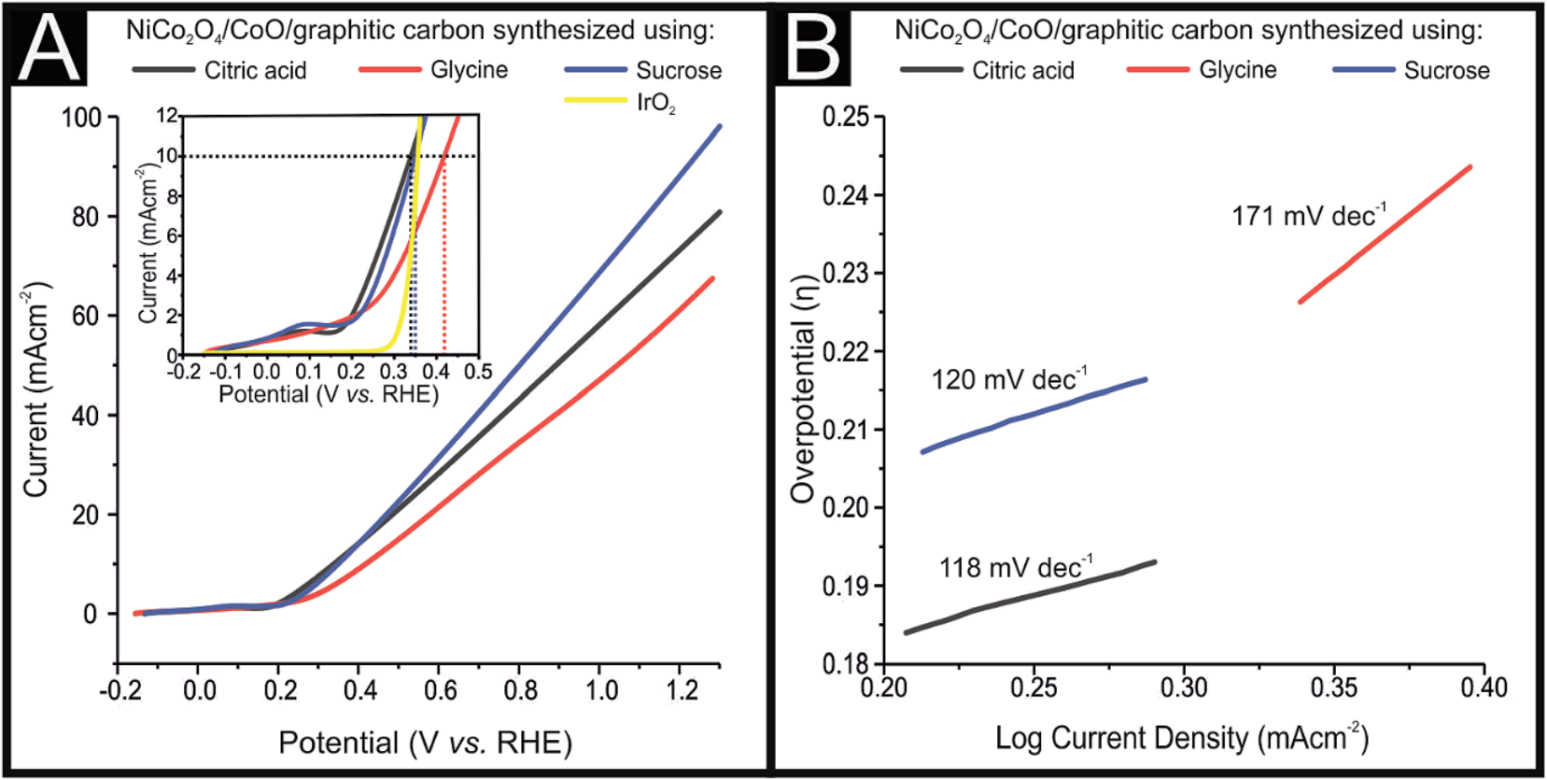
(A) Linear sweep voltammetry (LSV) showing the overpotential of the NiCo_2_O_4_/CoO/graphitic carbon composite modified SPE's prepared using citric acid (red), sucrose (black) and glycine (blue), in 1 M KOH. Scan rate: 20 mV s^−1^ (*vs.* RHE) (B) Tafel analysis; potential *vs.* log of current density for the faradaic region of the CV presented in (A).

The NiCo_2_O_4_/CoO/graphitic composites prepared using citric acid, sucrose and glycine were explored towards the OER. The electrocatalytic performance of the NiCo_2_O_4_/CoO/graphitic carbon composites were further examined using linear sweep voltammetry (LSV) in a solution of 1 M KOH with the obtained LSVs being displayed in Fig. 5(A). The LSV curves of the NiCo_2_O_4_/CoO/graphitic carbon composites prepared using citric acid and sucrose, in Fig. 5(A), indicate the presence of an oxidation peak at +0.13 V *vs.* RHE (inset of Fig. 5(A)). The observed peak at +0.13 V is a result of the oxidation of Co^3+^ to Co^4+^.^27^ This oxidation of Co^3+^ to Co^4+^ suggests that the Co^3+^ present at the octahedral site is responsible for the onset of the OER.^28^ No oxidation peak is observed for the NiCo_2_O_4_/CoO/graphitic carbon composite prepared using glycine, moreover, the mechanism for the OER using this composite is not clear and requires further study. The dramatic increase in current density after +0.14 V *vs.* RHE in all three cases demonstrates the onset of the OER. The inset of Fig. 5(A) shows the overpotential required to produce 10 mA cm^−2^ for the NiCo_2_O_4_/CoO/graphitic carbon composites prepared from citric acid, sucrose and glycine, which were found to be 323 mV, 344 mV and 408 mV, respectively. The overpotential observed for the composite prepared from citric acid is less electropositive compared to that of the composite prepared from sucrose and glycine, indicative of a lower activation energy for the OER to progress. The results presented above suggest that the presence of graphitic carbon enhances the conductivity of the electrolyte and thereby enhances the charge and mass transfer during the OER at the surface of the metal oxide.^29^ The overpotential required to produce a current density of 10 mA cm^−2^ exhibited by the NiCo_2_O_4_/CoO/graphitic (323 mV) is less electropositive than the overpotential required by traditionally employed electrocatalysts IrO_2_ (340 mV) and RuO_2_ (350 mV) and Au/NiCo_2_O_4_ nanorod array (360 mV). The LSV for an IrO_2_ can be seen within the inset of Fig. 5. Additionally, of all the studies reported in Table 1 the NiCo_2_O_4_/CoO/graphitic composite prepared using citric acid, displayed the best performing electrochemical activity in terms of OER onset potential, in fact, it displayed the smallest overpotential that the authors of this manuscript have found to date within literature.^22,28,30–32^ The citric acid NiCo_2_O_4_/CoO composite is therefore a viable alternative to precious metals as the anodic material within water electrolysers. It is important to note that whilst the citric acid NiCo_2_O_4_/CoO composite variant displayed the lowest overpotential post 0.45 V (*vs.* RHE) the NiCo_2_O_4_/CoO composite produced using sucrose displayed the largest achievable current density.

**Fig. 6:**
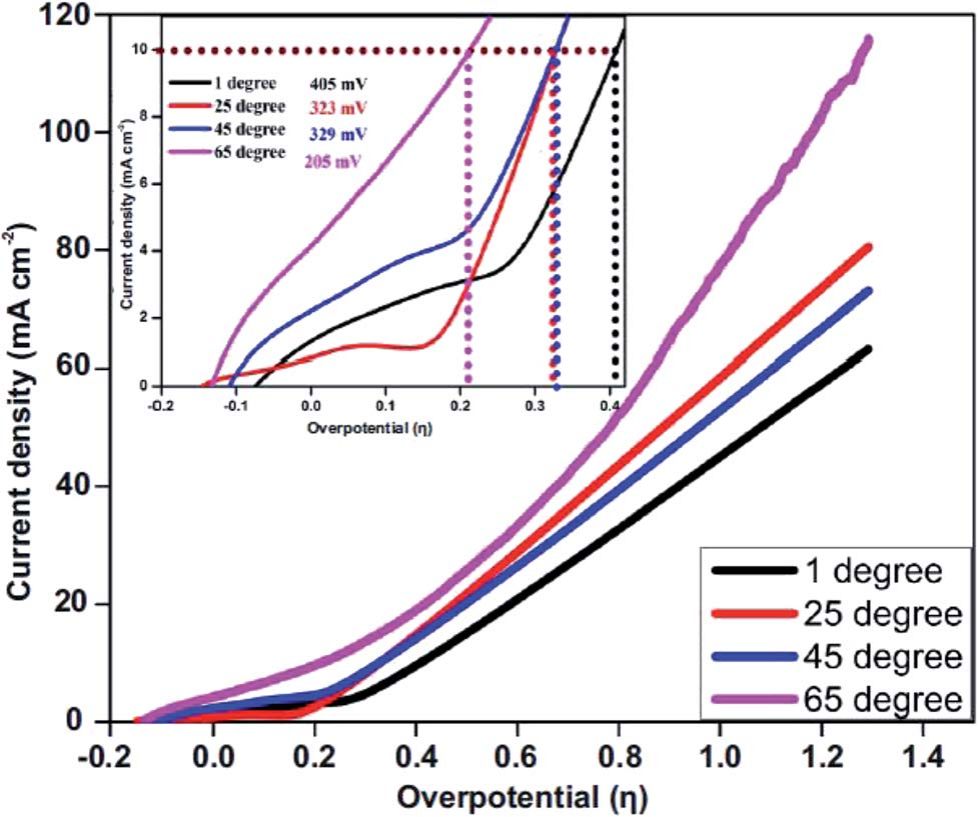
Effect of temperature on OER catalysis (overpotential) exhibited by the NiCo_2_O_4_/CoO/graphitic carbon composite prepared using citric acid at (black) 274, (red) 300, (blue) 320 and (purple) 340 K.

The effect of temperature on the electrocatalytic behavior of the NiCo_2_O_4_/CoO/graphitic carbon composites on OER performance is displayed in Fig. 6. Using *η* and the obtained Tafel values as a function of temperature, the catalytic performance of the composite prepared with citric acid within a varied temperature range was measured towards the OER in 1 M KOH solution using LSV and the corresponding anodic current response. The overpotential required to produce 10 mA cm^−2^ of current density at temperatures of 274, 300, 320 and 340 K was found to be 405 mV, 329 mV, 326 and 205 mV, respectively, suggesting the OER performance increased monotonically with temperature.^33^ Thus, at higher temperatures enhanced OER activity is observed, accordingly, the NiCo_2_O_4_/CoO/graphitic carbon composite is potentially a good catalyst with the utilization of the infrared range of the solar spectrum for water oxidation.

The OER kinetics of the NiCo_2_O_4_/CoO/graphitic carbon composites derived from citric acid, sucrose and glycine were assessed by Tafel analysis, as shown in Fig. 5(B), Tafel analysis demonstrates the co-relation between over potential (*η* and current density (log *J*) wherein it is used to deduce the reaction mechanism by which the OER progresses. The value of the Tafel slope was determined from the polarization curves and the corresponding Tafel values of the composites derived from citric acid, sucrose and glycine were 118, 120 and 171 mV dec^−1^, respectively. Analysis of the Tafel values can be utilized to determine the rate determining step for the OER reaction mechanism (mechanism displayed below).^21,34–38^

1 M + OH^−^ → e^−^ + MOH_(adsorption)_

2 MOH + OH^−^ → e^−^ + H_2_O + MO

3 MO + OH^−^ → e^−^ + MOOH

4 MOOH + OH^−^ → H_2_O + e^−^ + MOO

5 MOO → O_2_ + M

where M represents the active site. It is reported that the theoretical Tafel values of 120, 30 and 20 mV dec^−1^ correspond to the rate-determining steps for OH^−^ adsorption, O–H bond breaking and O_2_ desorption, respectively. The Tafel slope values of the NiCo_2_O_4_/CoO/graphitic carbon composites derived from citric acid and sucrose are 118 and 120 mV dec^−1^, suggesting the rate determining step is OH^−^ adsorption. The Tafel slope comparison suggests that the OER kinetics of the composite derived from citric acid is faster than that of the composite derived from sucrose and glycine. The faster kinetics may be due the presence of graphitic carbon and high lateral size with thin and flat surfaces relating to surface morphology, which increase the electronic conductivity (increased number of electroconductive pathways) and active sites to enhance the charge and mass transfer process.^39^ Thus, the low overpotential and modest Tafel value of the NiCo_2_O_4_/CoO composite derived from citric acid proves the composite to be an efficient catalyst for water oxidation.

**Fig. 7:**
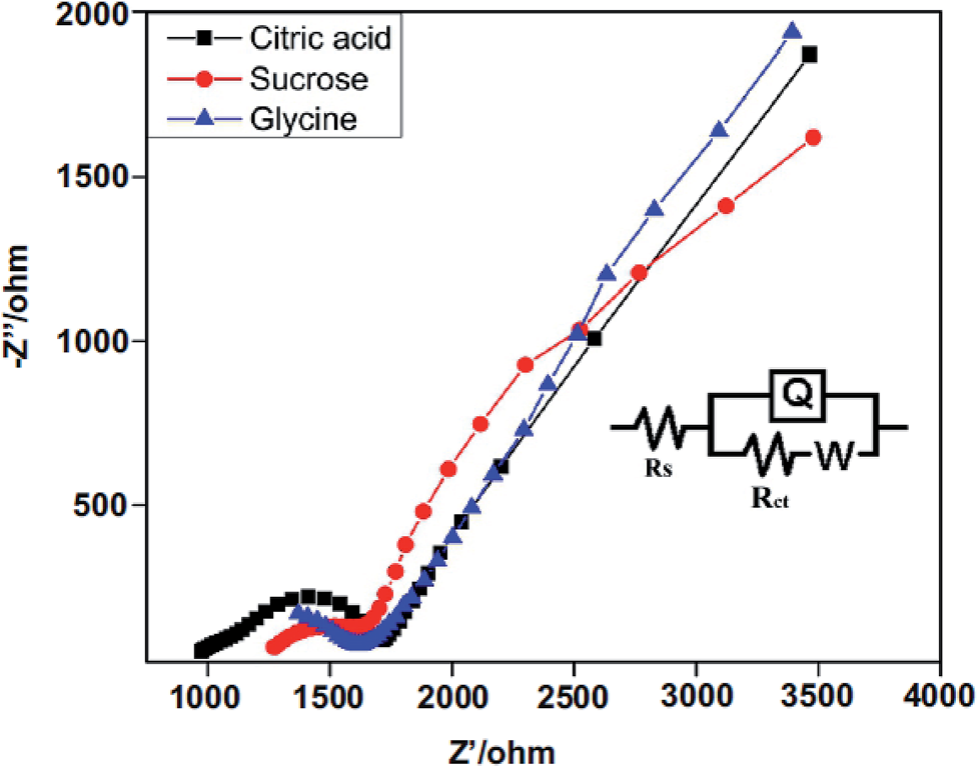
Electrochemical impedance Nyquist plots of fresh NiCo_2_O_4_/CoO/graphitic carbon composite in 1 M KOH solution, frequency range 0.01 Hz to 100 kHz; inset: equivalent circuit.

In order to understand the excellent OER electrocatalytic performance of the NiCo_2_O_4_/CoO composite derived from citric acid, electrochemical impedance spectra (EIS) were recorded between the frequency range 0.01 Hz and 100 kHz. The electrochemical performance of the composite is affected by charge transfer resistance (*R*_ct_) at the electrode/electrolyte interface. Thus, EIS measurements were carried out to investigate the *R*_ct_. Fig. 7 depicts the Nyquist plots of fresh NiCo_2_O_4_/CoO/graphitic carbon composites and the measured impedance spectra were analyzed using Zsimpwin software by fitting with an electrical equivalent circuit. An equivalent circuit is composed of *R*_ct_, constant phase element *Q*, and Warburg impedance (*W*) corresponds to different electrochemical processes that occur at the electrode/electrolyte interface. The Nyquist plot consists of an intercept and straight sloping line at high and low frequency regions corresponding to the *R*_ct_ and *W* respectively.^40^ The NiCo_2_O_4_/CoO/graphitic carbon composite derived from citric acid exhibits charge transfer resistance (Ω) of *ca.* 920.34 Ω while *ca.* 1242.18 Ω and *ca*. 1353.94 Ω are observed for composites derived from sucrose and glycine, respectively. The higher conductivity/lower resistivity of the composite derived from citric acid is consistent with the LSV results presented in Fig. 4(A) and suggest the sucrose NiCo_2_O_4_/CoO/graphitic carbon composite is the optimal electrocatalyst.

The stability and durability of a catalyst are important considerations if they are to be utilized within an industrial application. The stability and durability of the NiCo_2_O_4_/CoO/graphitic carbon composites were studied using 1000 repeated LSVs at a scan rate of 20 mV s^−1^ in 1.0 M KOH, where the retention of the current density after 100, 200, 500 and 1000 cycles is summarized in Table 2. Table 2 shows that the NiCo_2_O_4_/CoO/graphitic carbon composite derived from citric acid displays an excellent stability comparable to the composite derived from sucrose and glycine. It is observed that the NiCo_2_O_4_/CoO/graphitic carbon composites derived from citric acid and sucrose exhibit good current retention of about 91.8 and 90.9% after 200 cycles, respectively. Furthermore, current density retention of 63.2 and 61.6% has been observed after 200 cycles for the NiCo_2_O_4_/CoO/graphitic carbon composites derived from citric acid and sucrose, respectively, whereas, a 56% current density retention for the composite derived from glycine is indicative of poorer stability. Thus, stability for the NiCo_2_O_4_/CoO/ graphitic carbon composite varied in the order of citric acid > sucrose > glycine. In addition to the cyclic stability test, chronoamperometry was performed on the composites derived from citric acid, sucrose and glycine, carried out at an applied potential of 0.7 V *vs.* Ag/AgCl in 1 M KOH for a period of 10 hours, the corresponding results are shown in ESI Fig. S2.† The current density for all composites increases initially and then becomes almost stable after 2 hours. After 10 hours of cycling, the NiCo_2_O_4_/CoO/graphitic carbon composite derived from citric acid exhibits a current density retention of 93%, demonstrating excellent stability. Under the same conditions, the composites derived from sucrose and glycine display a current density retention of 90.71%, and 90% respectively. Thus, the stability of the proposed electrodes herein exceeds that of the benchmarked IrO_2_ and RuO_2_ based electrodes previously reported within literature.^41–44^

**Table 2:** The stability of the NiCo_2_O_4_/CoO/graphitic carbon composite derived from citric acid, sucrose and glycine^a^

NiCo_2_O_4_/CoO/graphitic carbon composite	Current density retention after 100 cycles	Current density retention after 200 cycles	Current density retention after 500 cycles	Current density retention after 1000 cycles
Citric acid	99.2%	91.8%	63.2%	38.40%
Sucrose	98.6%	90.9%	61.6%	33.8%
Glycine	84.8%	56.6%	—	—

a Value not recorded.

The number of moles of O_2_ generated per second per mole by the NiCo_2_O_4_/CoO/graphitic carbon composite at *η*_10_, is expressed using turn over frequency, calculated using current density obtained from LSV.^45^ The value of TOF for the NiCo_2_O_4_ catalyst is found to be 1.53 × 10^−2^ s^−1^, which is close to the TOF value for NiCo_2_O_4_/NiO (1.4 × 10^−2^ s^−1^)^46^ and superior to that of the TOF value reported for NiFe_2_O_4_ (5.74 × 10^−4^ s^−1^) and M_*x*_Ni_1−*x*_Fe_2_O_4_ (0.7–1.87 × 10^−4^ s^−1^).^34^ This indicates that the NiCo_2_O_4_/CoO/graphitic carbon composite can serve as an efficient and stable catalyst for water oxidation.

## 4. Conclusions

A rapid one-pot synthesis has been proposed to prepare NiCo_2_O_4_/CoO composites with varied additions of graphitic carbon using citric acid, sucrose and glycine. The NiCo_2_O_4_/CoO/graphitic carbon composite prepared with citric acid exhibits the optimal overpotential (*η*_10_), Tafel slope and TOF with values of 323 mV, 118 mV dec^−1^ and 1.53 × 10^−2^ s^−1^, respectively. The excellent electrochemical performance of the composite towards the OER could be related to the synergetic effect of graphitic carbon and the large lateral dimensions with thin and flat surface morphology. Moreover, the electrocatalytic performance towards the OER exhibited by the NiCo_2_O_4_/CoO/graphitic carbon composite prepared with citric acid is superior to the performance reported in previous studies using highly regarded electrocatalysts IrO_2_ and RuO_2_. It follows that the composite derived from citric acid is a potentially excellent electrocatalyst for overall water splitting applications given its significant electro-kinetic activity towards the OER. Adoption of the proposed method to explore other non-precious metal oxides to enhance the OER performance is underway.

## Conflicts of interest

There are no conflicts to declare.

## Supplementary Material

RA-009-C9RA05195C-s001
