# Supplementary material for: In situ addition of graphitic carbon into a NiCo2O4/CoO composite: enhanced catalysis toward the oxygen evolution reaction
Source: RSC Adv. 2019 Aug 12;9(43):24995–5002. doi: 10.1039/c9ra05195c (PMC9069886; doi:10.1039/c9ra05195c)
Supplement: RA-009-C9RA05195C-s001 [file RA-009-C9RA05195C-s001.pdf]

Electronic Supplementary Material (ESI) for RSC Advances.
This journal is © The Royal Society of Chemistry 2019

Electronic Supporting Information (ESI) for the following publication:

In Situ addition of Graphitic Carbon into a $NiCo_2O_4$/CoO composite: its Enhanced Catalysis toward the Oxygen Evolution Reaction

Srinivasa N, Shreenivasa L,[1] Prashanth S. A,[2] Jack P. Hughes,[3,4] Samuel J. Rowley-Neale,[3,4] Craig E Banks [3,4]* and Ashoka S[1*]

[1]: Department of Chemistry, School of Engineering, Dayananda Sagar, University, Bangalore, India

[2]: PG Department of Chemistry, KLE's PC Jabin Science College, Hubballi, India

[3]: Faculty of Science and Engineering, Manchester Metropolitan University, Chester Street, Manchester M1 5GD, UK.

[4]: Manchester Fuel Cell Innovation Centre, Manchester Metropolitan University, Chester Street, Manchester M1 5GD, UK.

*To whom correspondence should be addressed.

Email: c.banks@mmu.ac.uk; S.Rowley-Neale@mmu.ac.uk; Tel: ++(0)1612471196; Fax: ++(0)1612476831

Website: www.craigbanksresearch.com

ESI Figure 1. SEM images of the $NiCo_2O_4$/CoO/graphitic carbon composite prepared at 500 °C/3 minutes using (a, b) citric acid, (c, d) sucrose, (e, f) glycine.

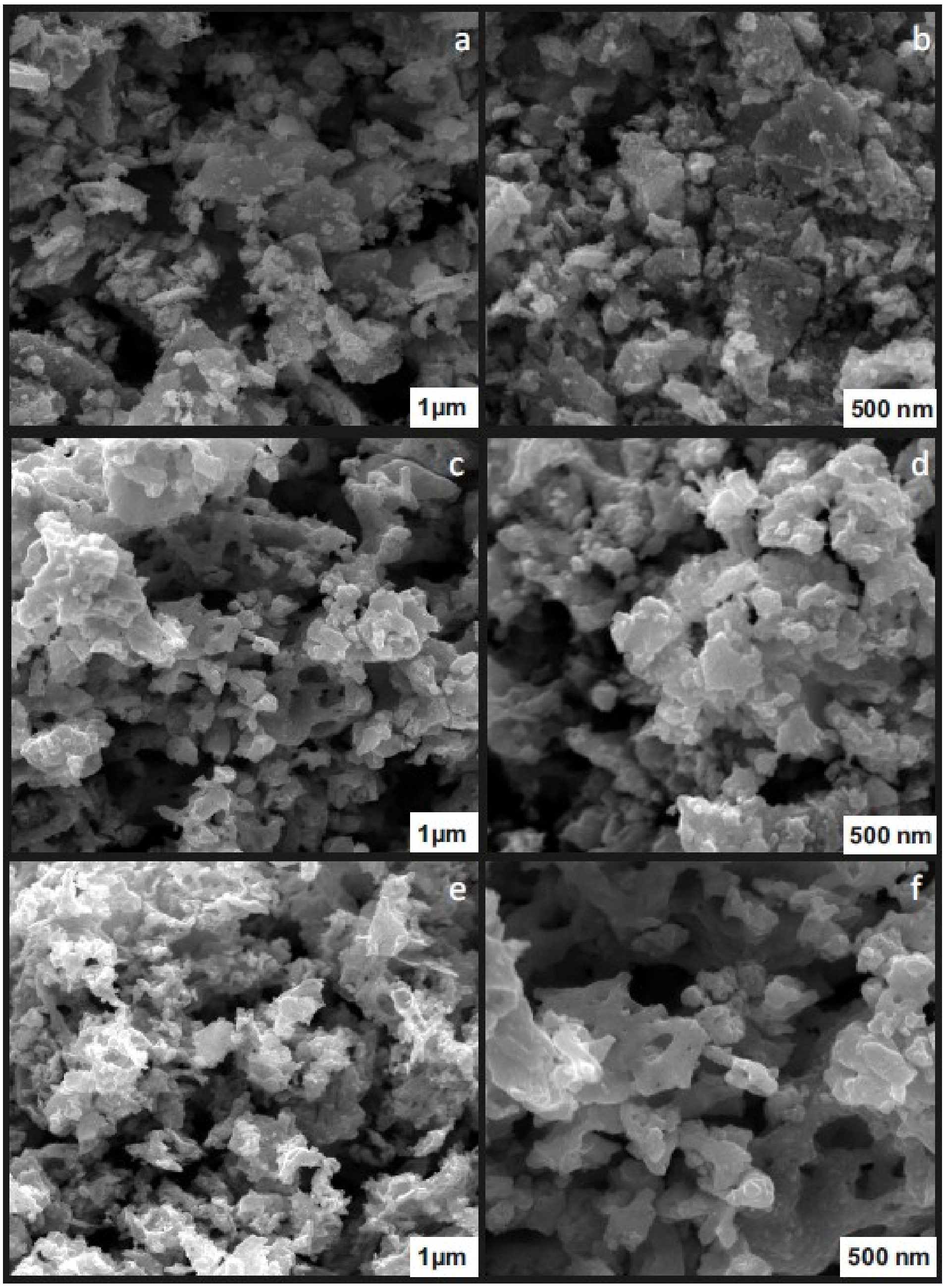

ESI Figure 2. Chronoamperometry stability curves of $NiCo_2O_4$/CoO/graphitic carbon composite derived from (a) citric acid, (b) sucrose and (c) glycine in applied potential of + 0.7 V in 1M KOH in the period of 10 hours.

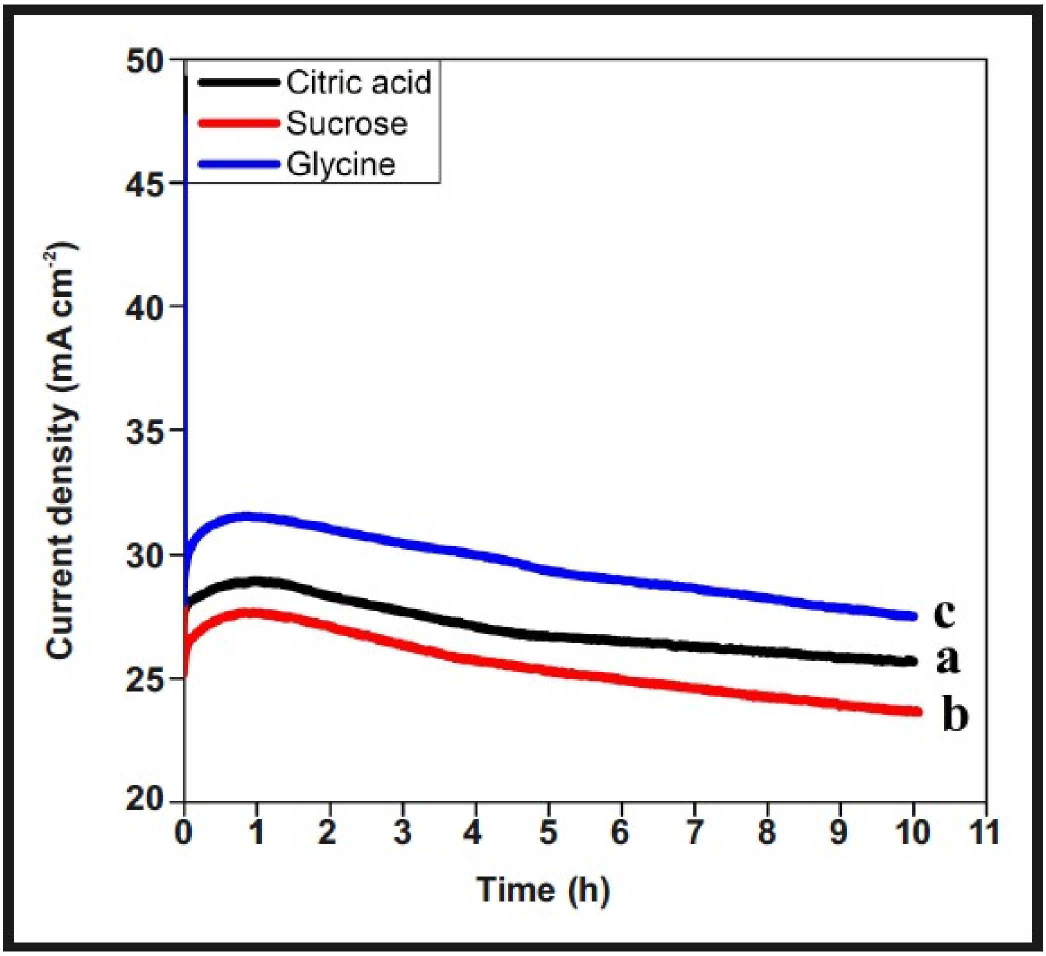

Electrochemical calculations

Conversion of the potential measured *vs.* Ag/AgCl to RHE electrode

The potential measured *vs.* the Ag/AgCl electrode, was converted to the reversible hydrogen electrode (RHE) scale according to the Nernst equation [1-3]

$E_{RHE} = E_{Ag/AgCl} + 0.059 * 14 + 0.196$

The overpotential (η) was calculated according to the following equation [1-3]

$\eta\ (V) = E_{RHE} - 1.23\ V$

Turn over frequency

Turn over frequency (TOF) was calculated according to the following equation:

TOF= jS/4Fn

where j is the measured current density at η_{10}, S is the working electrode area (cm^2), F is the Faraday constant, 4 is the number of electrons involved in the OER and n is the number of moles of the catalyst present in the working electrode. [1, 2].

1. V. Maruthapandian, M. Mathankumar, V. Saraswathy, B. Subramanian and S. Muralidharan, *ACS Appl. Mater. Interfaces*, 2017, **9**, 13132-13141.
2. C. Chang, L. Zhang, C.-W. Hsu, X.-F. Chuah and S.-Y. Lu, *ACS Appl. Mater. Interfaces*, 2018, **10**, 417-426.
3. H. Shi and G. Zhao, *J. Phys. Chem. C*, 2014, **118**, 25939-25946.
